# Highly sensitive VOC detectors using insect olfactory receptors reconstituted into lipid bilayers

**DOI:** 10.1126/sciadv.abd2013

**Published:** 2021-01-13

**Authors:** Tetsuya Yamada, Hirotaka Sugiura, Hisatoshi Mimura, Koki Kamiya, Toshihisa Osaki, Shoji Takeuchi

**Affiliations:** 1 Artificial Cell Membrane Systems Group, Kanagawa Institute of Industrial Science and Technology, 3-2-1 Sakado, Takatsu-ku, Kawasaki, Kanagawa 213-0012, Japan.; 2 Division of Molecular Science, Graduate School of Science and Technology Gunma University, 1-5-1 Tenjin-cho, Kiryu city, Gunma 376-8515, Japan.; 3 Institute of Industrial Science, The University of Tokyo, 4-6-1 Komaba, Meguro-ku, Tokyo 153-8505, Japan.; 4 Department of Mechano-Informatics, Graduate School of Information Science and Technology, The University of Tokyo, 7-3-1 Hongo, Bunkyo-ku, Tokyo 113-8656, Japan.; 5 International Research Center for Neurointelligence (WPI-IRCN), The University of Tokyo Institutes for Advanced Study (UTIAS), The University of Tokyo, 7-3-1 Hongo, Bunkyo-ku, Tokyo 113-0033, Japan.

## Abstract

This paper reports a volatile organic compound (VOC) sensor based on olfactory receptors that were reconstituted into a lipid bilayer and used in a specifically designed gas flow system for rapid parts per billion (ppb)–level detection. This VOC sensor achieves both rapid detection and high detection probability because of its gas flow system and array design. Specifically, the gas flow system includes microchannels and hydrophobic microslits, which facilitate both the introduction of gas into the droplet and droplet mixing. We installed this system into a parallel lipid bilayer device and subsequently demonstrated parts per billion–level (0.5 ppb) detection of 1-octen-3-ol in human breath. Therefore, this system extends the various applications of biological odorant sensing, including breath diagnosis systems and environmental monitoring.

## Teaser

A highly sensitive odorant sensor was developed by using insect olfactory receptors reconstituted into a lipid bilayer membrane.

## INTRODUCTION

Olfactory receptors (ORs) in living organisms can recognize various volatile organic compounds (VOCs) with a level of detection corresponding to a single molecule (*1*, *2*). Compared with the current VOC sensors using artificial materials, the receptors in living systems are far superior in selectivity and sensitivity. Recently, a biohybrid sensor that directly uses ORs as sensing elements has drawn attention for providing highly selective and sensitive molecular-level sensing of VOCs (*3*).

Biohybrid sensors using ORs are generally categorized into two types: cell-based (*4*–*6*) and cell-free (*7*, *8*). In cell-based sensors, odorant signals are detected as electrical signals or as changes in the fluorescence intensity caused by intracellular reactions. On the other hand, in cell-free sensors, ORs can be reconstituted into a lipid bilayer, and the response of the receptors is analyzed at the molecular level by electrophysiological recordings; typically, the lipid bilayer can be easily formed by contacting two droplets covered with a lipid monolayer (*9*). These cell-free sensors use a purified OR that reduces the complex interactions and unnecessary biochemical reactions in the sensing process (*10*); therefore, the cell-free sensors are expected to have improved reproducibility, quantification, and storage stability, compared with their cell-based counterparts. Although VOC sensors using ORs have been reported with high specificity, the sensitivity often remained limited because of the low solubility of VOCs (*8*, *11*). Therefore, the efficient introduction of VOCs into aqueous droplets and the transport of VOCs to ORs are prominent challenges in developing highly sensitive VOCs sensors.

**Fig. 1:**
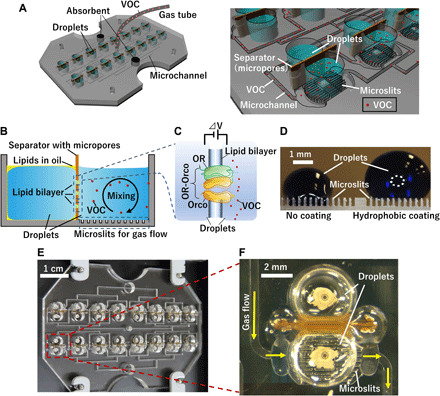
Overview of the proposed VOC sensor. (**A**) Schematic of the lipid bilayer array with the gas flow system. (**B**) Schematic of the double-well cross section. (**C**) Schematic of sensing mechanism using OR-Orco in a lipid bilayer. (**D**) Side view of aqueous droplets on microslits with and without the hydrophobic coating. VOC gas was introduced through the microslits underneath the droplets. (**E**) Photograph of the array device with the gas flow system. (**F**) Photograph of the double well after droplet addition. Photo credit: Tetsuya Yamada, Kanagawa Institute of Industrial Science and Technology.

In this study, we develop a cell-free VOC sensor combined with a gas flow system that achieves high detection probabilities at low VOC concentrations (Fig. 1, A to C). A lipid bilayer–forming droplet is placed on hydrophobic microslits (Fig. 1D), which create a gas flow channel underneath the droplet. Continuous gas flow through the channel enables VOCs to be quickly introduced into the droplet. Furthermore, the shear stress at the liquid-gas interface induces convection in the droplet, which further facilitates the transport of VOCs to the OR. In addition, an array of lipid bilayers is designed to enhance the detection probability. As a sensing receptor, we used an OR with an OR co-receptor (OR-Orco), which is an insect receptor forming a ligand-gated ion channel. As a practical example, we demonstrate the detection of 1-octen-3-ol (octenol) at the parts per billion (ppb) level, which is a biomarker in human breath, by using a VOC sensor consisting of OR-Orco reconstituted in a lipid bilayer.

## RESULTS

### Characterization of droplets stirred by continuous gas flow through microslits

**Fig. 2:**
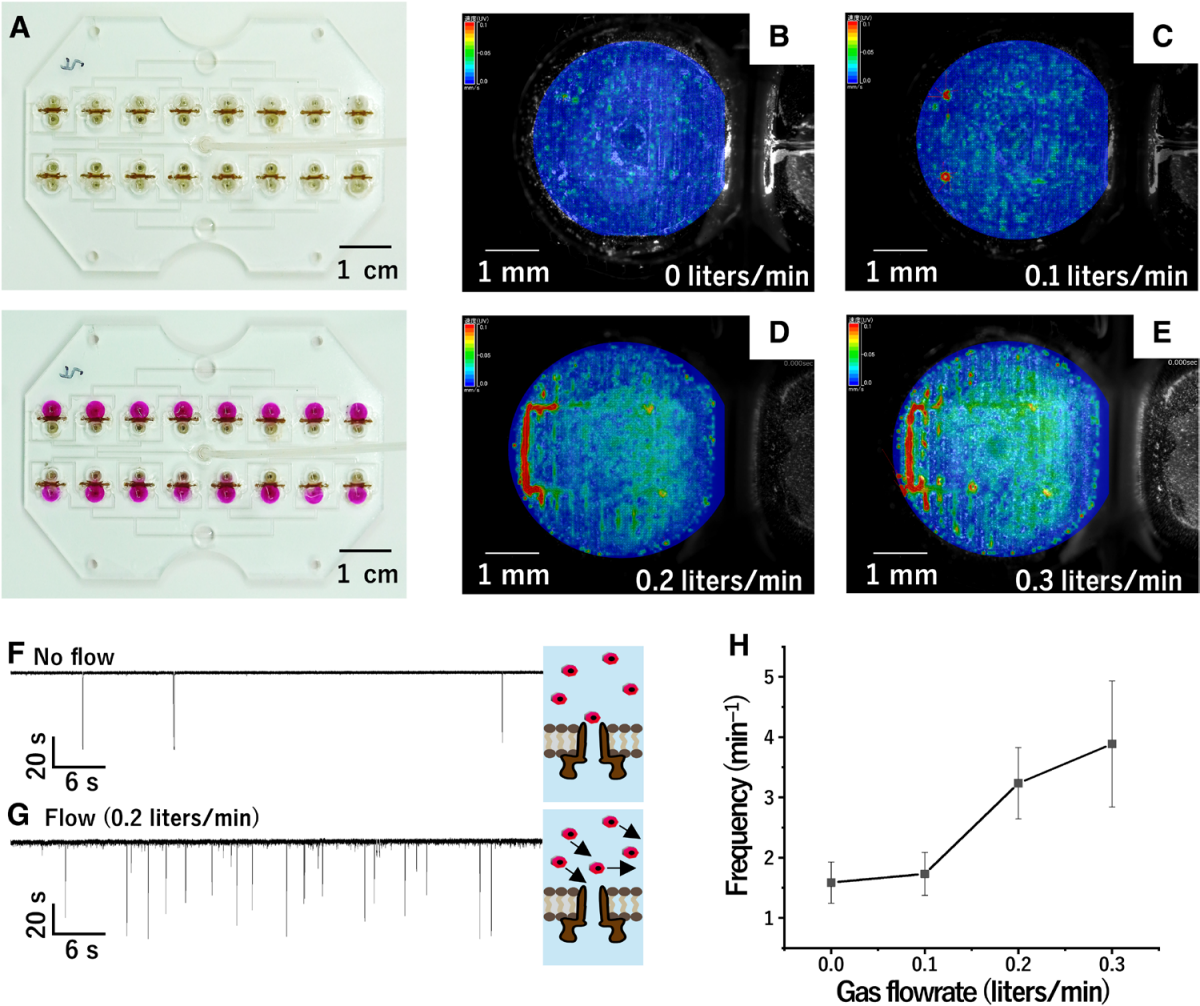
Characterization of gas flow system using microslits underneath droplets. (**A**) Photographs demonstrating the simultaneous introduction of ammonia gas into the 16 double wells through the microchannel. Top: Before the gas introduction. Bottom: After the gas introduction. Phenolphthalein was added to the droplets as a pH indicator for the introduction of ammonia gas. (**B** to **E**) Particle image velocimetry analysis of particle images with N_2_ gas flow rates of 0 liters/min (B), 0.1 liters/min (C), 0.2 liters/min (D), and 0.3 liters/min (E) (particle size, 10 μm). (**F** and **G**) Current-time traces of the α-hemolysin nanopore with blockers applied, without (F) and with (G) N_2_ gas flow (flow rate, 0.2 liters/min). (**H**) Relationship between blocking frequency and N_2_ gas flow rate. Error bars represent standard error (*N* = 4). Photo credit: Tetsuya Yamada, Kanagawa Institute of Industrial Science and Technology.

To confirm the formation of gas flow channels underneath the droplets, a droplet containing blue ink (for visualization) was placed on the microslits. Figure 1D shows a side-view image of this droplet on the hydrophobic microslits. The droplet did not intrude into the microslits, suggesting that the gas flow channel was successfully formed in the microslits. Ammonia gas was introduced to the microslits to confirm that gas was effectively introduced into droplets; a pH-sensitive dye, phenolphthalein, was dissolved in the droplets (*12*). The droplets became pink after ammonia gas was introduced (Fig. 2A), indicating that the gas was successfully introduced into the droplet through the microslits/microchannels. Movie S1 shows the time course of the color change, demonstrating that the color change simultaneously occurs over all droplets; this result is due to the microchannel pattern, which has a symmetrical design to generate a uniform flow rate throughout the channels. This uniform flow rate is important for parallel recording using multichannel sensor units.

To investigate the influence of the gas flow on the droplet, we added microbeads (diameter: 45 μm) to the droplet and observed their movement under a microscope (movie S2). The microbead movement was initially slow without gas flow. When N_2_ gas flow was initiated, the microbeads began to circulate (movie S2), clearly indicating that the droplet was mixed by the gas flow; shear stress was likely applied to the gas-liquid interface. This phenomenon is similar to that in a previous study that demonstrated droplet mixing caused by shear stress between oil and an aqueous droplet (*13*). We used particle image velocimetry (PIV) to analyze the microfluidic flow (Fig. 2, B to E) and investigate the relationship between fluidic convection and gas flow rate. The results indicated that the velocity distribution pertains to gas flow rate; the color of the images in Fig. 2 (B to E) represents particle velocity. We found that particle velocity increased against the gas flow rate. In the case of no gas flow, particles with a velocity of higher than 0.02 mm/s constituted 4% of all the particles in the droplet. On the other hand, at the gas flow rates of 0.1, 0.2, and 0.3 liters/min, particles with a velocity of higher than 0.02 mm/s accounted for 8, 44, and 51%, respectively, of all the particles. This shift in the velocity distribution to a higher particle velocity with the increasing gas flow rate shows that the higher gas flow rate provides more vigorous mixing in the droplet.

To confirm the mixing effect on the response of the cell-free sensor, we analyzed the frequency of binding between the membrane proteins and ligands under mixing caused by the gas flow. In the experiment, α-hemolysin (αHL) and heptakis(6-*O*-sulfo)-β-cyclodextrin were used as a nanopore and a blocker, respectively (*14*). Figure 2 (F and G) shows the transmembrane current with and without mixing, respectively. The blocker was added in the droplet, and the nanopore was reconstituted in a lipid bilayer. The spike-like current signals directed downward are attributed to binding events between the blocker and the αHL nanopore (*15*), whereas the current recovered to the initial level is attributed to dissociation events. Clearly, the frequency of the signals increased with N_2_ gas flow compared with the frequency without gas flow (Fig. 2, F and G). The signal frequency shown in Fig. 2H increased from 1.6 to 3.9 min^−1^ when the N_2_ gas flow rate increased from 0 to 0.3 liters/min. This trend clearly suggests that N_2_ gas flow, which manifests as mixing in the droplet, enhances the signal frequency, providing a useful approach for developing cell-free sensors for rapid detection. However, 0.3 liters/min or higher flow rates could blow the droplet away, causing lipid bilayer rupture; hereinafter, we thus use flow rates under 0.3 liters/min. These results suggest that the gas flow enhances the signal frequency, which is important for sensor response.

In addition to the signal frequency, the dwell time and blockade current levels were analyzed (fig. S1). The dwell time represents the time for which a blocker remains over an αHL nanopore (time between the binding and the dissociation event), and the blockade current levels indicate the value of the current drop caused by a binding event. Overall, the dwell time exhibits a negative correlation with N_2_ gas flow rate, whereas no significant impact is observed on the blockade current levels. Figure 2H and fig. S1 together confirm that the binding events at the molecular level are directly influenced by mixing in droplets. These results imply that the mixing promotes dissociation between the nanopore and the blocker.

### Detection of octenol using ORs reconstituted into arrayed lipid bilayers

**Fig. 3:**
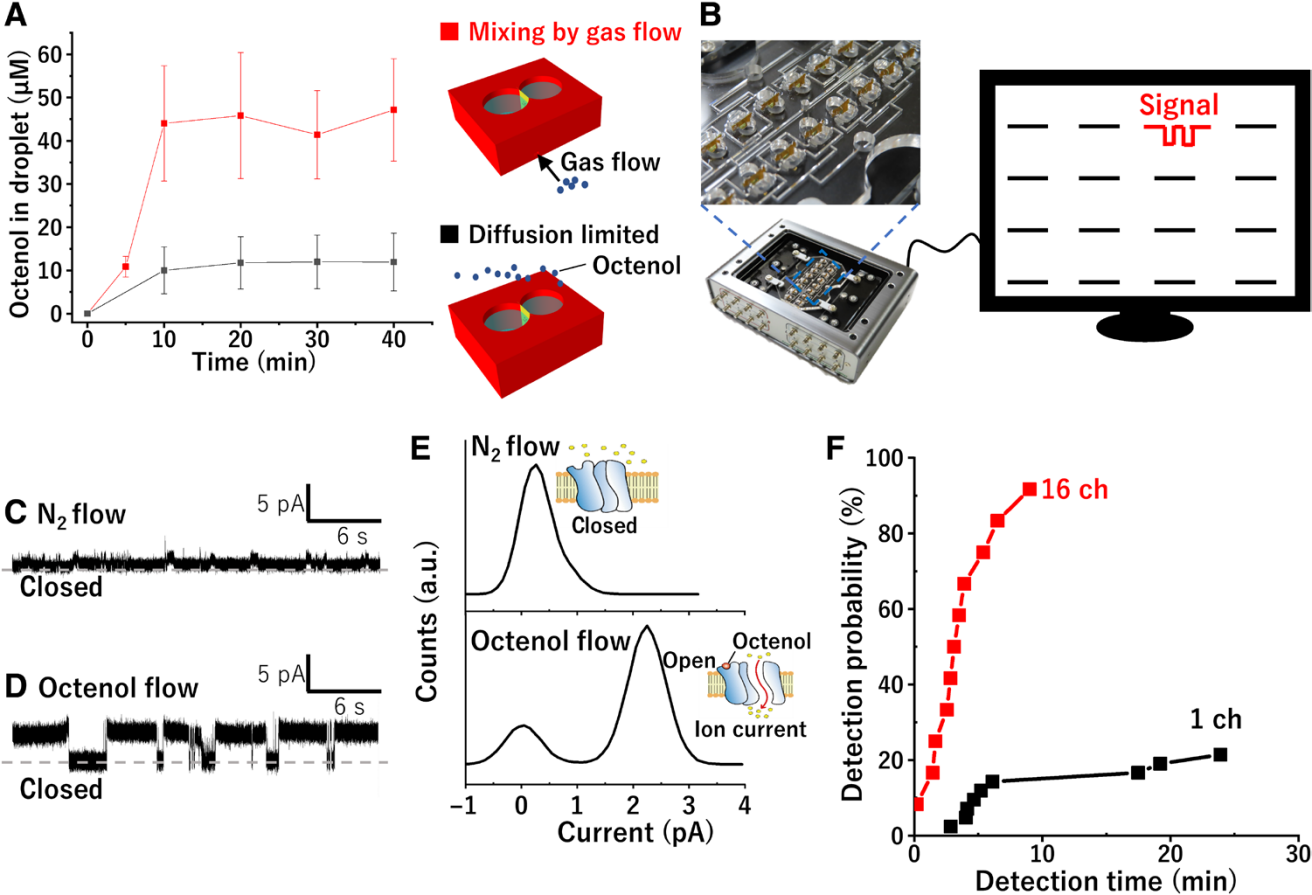
Detection of octenol gas using the developed device. (**A**) Relationship between the exposure time and the octenol concentrations in droplets either with (red) or without (black) the gas flow system. Schematics of the gas flow system and conventional gas exposure are included at the right of the plots. The error bars are the standard error (*N* = 6). (**B**) Schematic of the 16-channel gas flow device for parallel recordings. (**C** and **D**) Representative current traces of the OR-Orco in response to (C) N_2_ gas and (D) 5 ppm octenol gas. Carrier gas was N_2_. (**E**) Histograms of current traces for the OR-Orco in response to N_2_ gas and 5 ppm octenol shown in (C) and (D); a.u., arbitrary units. (**F**) Detection probability plotted against detection time for the 16-channel gas flow device and the single-channel device. Photo credit: Tetsuya Yamada, Kanagawa Institute of Industrial Science and Technology.

Octenol is a volatile metabolite present in human sweat (*8*, *16*), blood (*17*), and breath (*18*) and has low solubility. Initially, to detect octenol using OR-Orco, octenol introduction into the droplet was confirmed by gas chromatography measurements of the octenol concentration in the droplet (Fig. 3A). When the droplet was exposed to a gas flow of 5 ppm octenol with N_2_ carrier gas, the octenol concentration in the droplet increased with time until 10 min, at which point it plateaued at around 45 μM, suggesting that the octenol was successfully introduced and its concentration achieved a steady state. In addition, this system was able to remove octenol by introducing clean N_2_ gas flow (fig. S2). This N_2_ gas flow allows continuous detection via sample exchange. The octenol concentration was also investigated without the gas flow system by exposing the droplet to 5 ppm octenol. In this case, the octenol concentration slowly increased and did not reach a plateau even after 30 min, showing the superiority of our proposed gas flow system in terms of the response speed (Fig. 3A).

To confirm whether our developed sensor can detect octenol, we measured the electrical signals from yellow fever mosquito (*Aedes aegypti*) OR-Orcos, which are specifically sensitive to octenol (*19*). The OR-Orcos were obtained by a cell-free protein synthesis, and SDS–polyacrylamide gel electrophoresis (PAGE) analysis confirmed the formation of ORs and OR-Orcos (fig. S3). The OR-Orco yields were estimated to be 3.8 and 0.4 μg/ml for OR7 and OR8 using bovine serum albumin (BSA) standards, respectively. After the lipid bilayers were formed, parallel recordings of ionic current were initiated to measure the response of the receptors, as illustrated in Fig. 3B. Figure 3 (C and D) shows the representative current data under N_2_ gas and 5 ppm octenol, respectively, where open-closed signals were clearly observed with octenol gas flow. Low concentrations of octenol (0.5 or 5 ppb) were also introduced and open-closed signals were observed (fig. S4), showing that our sensor could detect octenol at parts per billion levels. In addition, acetone gas, which is a metabolite present in breath, was introduced as a negative control; no clear open-closed current steps were observed (fig. S5). To confirm that the open-closed current steps can be attributed to the OR-Orco, IR3535 inhibitor, which is well known to prohibit open-closed current signals, was added. As shown in fig. S6, the addition of IR3535 eliminated all open-closed current steps, suggesting that the open-closed signal observed in Fig. 3D can definitely be attributed to the OR-Orco. Furthermore, the current histogram shown in Fig. 3E (top) exhibits one peak under N_2_ gas flow, which represents the closed state of the OR-Orco. In Fig. 3E (bottom), two peaks representing the closed and open states were observed under octenol flow (5 ppm). These results suggest that the presence of octenol increases the probability of the open state of the OR-Orco.

Although our developed sensor using an OR-Orco was able to detect octenol, the sensing mechanism depends on stochastic events because only single-molecule dynamics were observed. To increase the detection probability, a device was designed with 16 channels. The multisensor unit achieved a detection probability of 92% in 10 min (Fig. 3F), detecting octenol in 11 of 12 samples (1 ppm octenol). In contrast, the single-channel device, without a gas flow system, exhibits a detection probability of only 14% in 10 min, detecting octenol in 6 of 42 samples at the same concentration of 1 ppm octenol. These results indicate that the 16-channel device with a gas flow system significantly improves the detection probability and detection time compared with a previously reported single-channel system (*8*).

### Detection of parts per billion–level octenol mixed in human breath

**Fig. 4:**
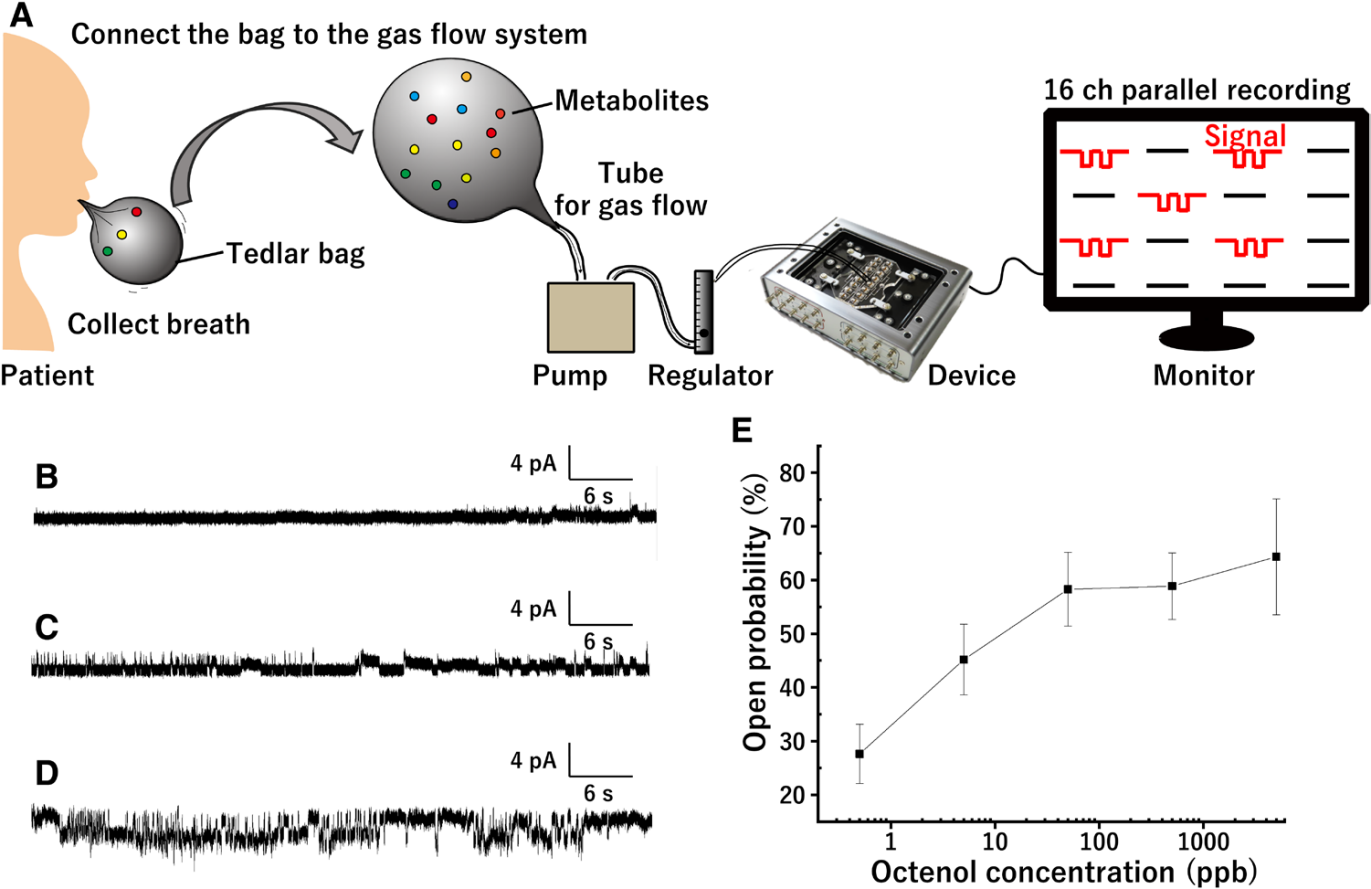
Specific detection of parts per billion–level octenol mixed in human breath. (**A**) Schematic of the detection process of octenol mixed in human breath using the proposed device. (**B**) Current traces for a human breath sample without octenol. (**C**) Current traces for the human breath sample with 0.5 ppb octenol and (**D**) 5 ppb octenol. (**E**) Open probability of the current signals plotted against octenol concentration mixed in the breath. The error bars show the standard error (*N* ≧ 6). Photo credit: Tetsuya Yamada, Kanagawa Institute of Industrial Science and Technology.

Octenol is also a human metabolite and a possible biomarker for cancer (*17*, *20*) and Coulomb disease. Octenol may cause neurodegeneration and cytotoxicity (*21*, *22*). It is therefore important to detect and distinguish between different metabolite biomarkers to detect diseases in the early stages. Testing human breath for specific biomarkers provides a convenient mechanism for disease diagnosis because it can be easily sampled, enabling cost-effective clinical diagnostic procedures (*20*). To demonstrate octenol detection in human breath, a gas flow setup was constructed as shown in Fig. 4A. First, human breath was collected by a Tedlar bag and transferred to the developed device (16-channel device) via the inhalation tube. A regulator maintains a constant flow rate of 0.2 liters/min. At this point, human breath was introduced to the developed device, and the 16 current signals were then monitored (movie S3). Figure 4 (B to D) shows the representative data of these current traces. Although the human breath contains about 3000 different metabolites (*20*), breath samples without octenol injected produced almost flat curves with a 13% open probability (Fig. 4B). In contrast, a breath sample with parts per billion–level octenol gas injected exhibited clear open signals even at 0.5 ppb (Fig. 4C), which cannot be detected when using normal gas chromatography without extraction (fig. S7), indicating that our proposed sensor is highly selective and sensitive.

In addition, the system can exchange samples for subsequent measurements, as shown in fig. S2. Octenol can be removed by airflow and then another gas can be introduced to initiate the next measurement. Figure S8 presents such continuous detection results. The frequency of closed states seen in red in the base current disappeared with the introduction of octenol but recovered under airflow, suggesting that the samples were successfully exchanged in preparation for the next measurement.

We next introduced (*S*)-1-octen-3-ol to investigate the relationship between open probability and octenol concentration and to quantify the open probability. Octenol exists in the form of two enantiomers, (*S*)-1-octen-3-ol and (*R*)-1-octen-3-ol, and the OR-Orco shows different responses to the enantiomers (*23*). We thus used (*S*)-1-octen-3-ol to quantify the open probability. Figure 4E shows that the open probability increased with the increasing octenol concentration and reached a plateau at around 500 ppb. This result again showed that our cell-free sensor was able to detect octenol in parts per billion levels.

## DISCUSSION

This work demonstrated a VOC sensor using an OR-Orco reconstituted into a lipid bilayer formed between two droplets (Fig. 1, B and C). This lipid bilayer is integrated on a chip with a gas flow system that introduces VOCs into the droplets and transports the VOCs to the OR-Orco to achieve high detection sensitivity. Because the gas flow system uses gas channels underneath droplets, the VOC was introduced into the droplets via mixing caused by gas flow, enabling parts per billion–level sensitivity for detecting octenol in human breath. The results indicate that the proposed cell-free sensor can exceed conventional limits of detection typically resulting from low VOC solubility.

For cell-based sensors, the complexity of living cells (*24*) creates unresolvable challenges, such as variability, dependence on cell conditions including the cell cycle and OR expression level, and instability caused by environmental changes. On the other hand, cell-free sensors are expected to present advantages such as low variability, good stability, longevity, and storage stability because the cell-free systems can activate biological machinery without living cells and eliminate unnecessary biological reactions. In addition, the membrane proteins can be synthesized using commercial cell-free protein synthesis methods using specific DNA, as shown herein, allowing us to easily form various types of ORs in an array.

Although highly sensitive VOC sensors based on ORs have been previously proposed for detecting VOCs dissolved in solutions (*7*, *25*), only a few studies have shown successful VOC detection directly from the gas phase (*4*, *8*, *10*). Therefore, the low solubility of VOCs limits the development of OR-based sensors. Our system facilitates the introduction of VOCs into droplets through continuous gas flow and droplet mixing. The mixing transports ligands to the membrane proteins (Fig. 2H), resulting in a high signal frequency. Moreover, we attribute the success of the highly sensitive VOC detector to the OR-Orco used in this work. Not only is OR-Orco a sensor that has a ligand-binding receptor (OR), but it also forms a unique complex with Orco, which can efficiently amplify the signal owing to its ion channel. In general, after one ligand molecule is bound to the ion channel, the ion channel can generate pA-level ionic flow through the cell membrane, meaning that the ion channel of the OR-Orco can amplify a single molecular signal to 10 million molecules per second.

In addition to designing the system for introducing VOCs into droplets, we arranged the lipid bilayers into an array to improve the detection probability. A single lipid bilayer exhibits low detection probability (Fig. 3F), which is attributed to the low OR reconstitution efficiency (*26*) and the stochastic characteristics of the binding between the OR and ligands (*27*). The lipid bilayer array design is a promising approach for improving detection probability. The developed gas flow system is also compatible with the array design by allowing the gas to reach the lipid bilayer arrays through microchannels. Moreover, the array design would be suitable to reconstitute different OR-Orcos into each arrayed lipid bilayer. There are several different types of insect ORs whose genes have been already identified (*28*). When using multiple ORs reconstituted in the sensor through cell-free protein synthesis, it would be possible to recognize more complex mixtures of VOCs including several biomarkers in human breath/urine. Therefore, the developed system using OR-Orco can be a powerful platform for disease diagnosis based on the detection of specific metabolites.

## MATERIALS AND METHODS

### Materials and reagents

*n*-Decane, 1-octen-3-ol, and wild-type αHL were obtained from Sigma-Aldrich (St. Louis, MO, USA). 1,2-Diphytanoyl-sn-glycero-3-phosphocholine (DPhPC), 1,2-dioleoyl-sn-glycero-3-phosphocholine (DOPC), 1,2-dioleoyl-sn-glycero-3-phospho-l-serine (DOPS), and 1,2-dioleoyl-sn-glycero-3-phosphoethanolamine (DOPE) were purchased from Avanti Polar Lipids Inc., (Alabaster, AL, USA). Heptakis(6-*O*-sulfo)-β-cyclodextrin heptasodium salt was purchased from Toronto Research Chemicals Inc. (Toronto, Canada). Chloroform, KCl, K_2_HPO_4_, CaCl_2_, MgCl_2_, sucrose, KH_2_PO_4_, activated charcoal, and LabAssay Phospholipid were purchased from Wako Pure Chemical Industries Ltd. (Tokyo, Japan). Hepes was purchased from Dojindo Molecular Technologies Inc. (Kumamoto, Japan). All aqueous solutions were prepared with ultrapure water from a Milli-Q system. Hydrophobic solvent (SFCOAT, SFE-B002H) and supercritical fluid extraction (SFE) solvent were provided by AGC SEIMI Chemical Co. Ltd. (Tokyo, Japan). A separator with 11 pores (diameter: 100 μm) was provided by a company. Ethyl 3-(*N*-butylacetamido)propanoate (IR3535) was purchased from Santa Cruz Biotechnology Inc. (Dallas, TX, USA). Poly(methyl methacrylate) (PMMA) substrates of 1- and 3-mm thickness were purchased from Mitsubishi Chemical Corporation (Tokyo, Japan). The Super X adhesive was purchased from CEMEDINE Co. Ltd. (Japan). Polystyrene microbeads, 45 μm in diameter, were purchased from Polysciences Inc. (Warrington, PA, USA), and polystyrene microbeads, 10 μm in diameter, were purchased from Kato Koken (Kanagawa, Japan). The cell-free translation system, PUREfrex 2.0, was obtained from GeneFrontier Corp. (Chiba, Japan). Oriole fluorescent gel stain was purchased from Bio-Rad Laboratories Inc. (California, USA).

### Device design and fabrication

A 16-channel device was assembled as shown in Fig. 1. The acrylic base of the device is composed of a micromachined 3-mm-thick PMMA substrate. The base has 16 pairs of double wells with a diameter of 4 mm and a depth of 3 mm, which were micromachined by a minimiller MM-100 (Modia Systems, Saitama, Japan). Microslits and microchannels were also micromachined on a 1-mm-thick PMMA substrate. The depth and width of the inflow microchannels were 0.7 and 0.5 mm, respectively. The distance from the inlet to microslits was 44.8 mm. The outflow microchannel depth and width were 0.7 and 0.5 mm, respectively. The distance from the microslits to the outlet was 36.8 mm. The 16 microchannels had a symmetrical design to equalize gas flow rates. The depth and spacing of the microslits were 0.5 and 0.3 mm, respectively. The microslits were micromachined using a mill (diameter: 0.2 mm). A pair of holes (diameter: 0.6 mm) was also drilled on the bottom substrate for placing the Ag/AgCl electrodes. These two PMMA substrates were bonded by thermocompression for 5 min at 100°C with an applied force of 25 N/cm^2^ and then cooled to room temperature gradually. Subsequently, the bottom surface of the wells was partially coated with Ag/AgCl paste for electrical measurements. Separators with 100-μm-diameter pores were inserted between the double wells and bound with an adhesive. The separator with micropores allows reproducible formation of stable lipid bilayers; the small diameter of the micropores stabilizes the lipid bilayer and reduces noise, as reported in our previous paper (*29*). The microslits were coated with a hydrophobic fluid (SFCOAT). The hydrophobicity of the surface allows good accessibility of gas. A 2.0-mm polytetrafluoroethylene (PTFE) tube (JR-T-082-M10, Shimazu) for gas flow was connected to the device with an adhesive. Adsorbents made of activated charcoal were placed at the microchannel outlets for capturing the VOCs emitted from the device, which could interfere with the measurements.

### Gas flow experiment using phenolphthalein for visualization of gas introduction

To demonstrate the introduction of gas into the 16 wells simultaneously, 0.1 w/v% of phenolphthalein was used as a pH-sensitive dye. To form a lipid bilayer, 5 μl of *n*-decane containing DOPC:DOPE (20 mg/ml) at a 3:1 (w/w) ratio was loaded to the wells with no microslits. Subsequently, 28 μl of buffer solution containing phenolphthalein was added to the wells with microslits, and 23 μl of buffer was added to the other wells. Subsequently, ammonia gas was collected from the gas-tight vial containing 1 ml of ammonia solution (25 to 28%) and was introduced into the device by a syringe (flow rate: 30 liters/min). Time-lapse images were obtained (interval: 5 s) and combined into a movie using ImageJ (NIH, USA).

### PIV for analyzing mixing under gas flow

The mixing by gas flow was imaged using microbeads (diameter: 45 μm). To form a lipid bilayer, 4.2 μl of DPhPC (20 mg/ml in *n*-decane) was added to the wells with no microslits. Then, 25 μl of buffer solution was added to the wells with microslits, and 21 μl of buffer was added to the other wells. The 1.0 M KCl buffer solution was adjusted to pH 7.0 with 10 mM phosphate buffer. The movement of the microbeads caused by gas flow was recorded by a microscope (VH-S5, KEYENCE, Japan). The gas flow rate was monitored by a flow meter. We used PIV to evaluate the mixing in the droplet. Tracers (diameter: 10 μm; Kato Koken, Japan) were dispersed at 0.1% solid concentration. The experimental system is composed of a 16-channel gas flow device, a visualization laser (PIV Laser G2000, Kato Koken, Japan), and a high-speed camera (k8-USB, Kato Koken, Japan). The laser irradiated a laser sheet (thickness: 2 mm) in a direction parallel to the gas flow device. The high-speed camera was placed above the gas flow device to obtain top-view images. We used four N_2_ gas flow conditions: 0, 0.1, 0.2, and 0.3 liters/min. The direct cross-correlation method was used for vector calculation using PIV analysis software (Flow Expert 2D2C, Kato Koken, Japan) (*30*). The differences in particle velocities were evaluated quantitatively depending on the particle position and the gas flow rate.

### Ion current measurement under gas flow for analyzing the effect of mixing on the signal from a membrane protein

We verified the effects of mixing in the droplet by monitoring the blocking events of s_7_βCD on αHL nanopores. To form a lipid bilayer, 4.2 μl of DPhPC (20 mg/ml in *n*-decane) was added to the wells with no microslits. Then, 25 μl of buffer solution containing 10 μM s_7_βCD was added to the wells with microslits, and 21 μl of buffer containing 1 nM αHL was added to the other wells. The 1.0 M KCl buffer solution was adjusted to pH 7.0 with 10 mM phosphate buffer. The ionic current passing through the lipid bilayer was recorded. The current was monitored with a sampling frequency of 5 kHz with a 1-kHz Bessel low-pass filter at a holding voltage of +60 mV. N_2_ gas was introduced from the inlet, and the gas flow rate was monitored by a flow meter. Under this condition, the αHL nanopores were spontaneously formed and generated stepwise ion current signals. The frequency of the blockade rate depending on the N_2_ gas flow rate was recorded. The blocking events were analyzed by software (pCLAMP Software, Molecular Devices, LLC, CA, USA) to determine dwell times and blockade current levels.

### Intake of octenol gas into the droplet forming lipid bilayer by gas flow

To form a lipid bilayer, 4 μl of *n*-decane containing DOPC:DOPE (20 mg/ml) at a ratio of 3:1 (w/w) was loaded to the wells with no microslits. Then, 28 μl of buffer solution [5 mM Hepes/KOH (pH 7.6) containing 0.8 mM CaCl_2_, 96 mM NaCl, 2 mM KCl, and 5 mM MgCl_2_] was added to the wells with microslits, and 24 μl of buffer was added to the other wells. After the formation of a lipid bilayer, octenol flow was started (5 ppm, at 0.25 liters/min) using a standard gas generator (PD-1B-2, Gastec, Japan), and the octenol concentration in the droplets was measured using a gas chromatography equipment (GC-2010 Plus, Shimadzu Corp., Japan). We evaluated the amount of octenol in the droplet based on a standard curve that was obtained using liquid samples with known concentrations of octenol. For comparison, we measured the octenol concentration without the gas flow system. In this case, the droplets forming the lipid bilayer are exposed (i.e., octenol gas fills up above the droplet); in addition, octenol concentration in the droplets was measured by gas chromatography. To estimate the concentration of octenol in human breath, an extraction tube (NeedlEx for fatty acid, Shinwa Chemical Industries Ltd.) was used.

### Preparation of OR-Orco

The lipids dissolved in chloroform were mixed with DOPC:DOPE:DOPS having a weight ratio of 5:3:2 (total lipid volume: 15 mg) and flushed with argon gas to form a lipid film in a glass tube. The tube was dried in a vacuum desiccator. Next, 300 μl of liposome inner solution [5 mM Hepes/KOH (pH 7.6) containing 0.8 mM CaCl_2_, 96 mM NaCl, 2 mM KCl, 0.2 M sucrose, and 5 mM MgCl_2_] was added to the lipid film to construct liposomes by hydration. The sample is vortexed and sonicated. To reduce the size of the liposomes, the solution was passed through a film extruder with pores of 100-nm diameter.

To obtain ORs (OR8) and OR co-receptor (OR7) proteins of the yellow fever mosquito (*A. aegypti*), we used a reconstituted cell-free translation system. OR and OR co-receptor DNA (OR:OR co-receptor = 1:3; total DNA: 2 μg) were added to the translation system (PUREfrex 2.0). We added 50 μl of liposomes into the cell-free translation system to form proteoliposomes. The incubation proceeded at a temperature of 25°C for 2 hours. Subsequently, the protein samples were centrifuged at 15,000 rpm at 20°C for 10 min. The supernatant was centrifuged using Amicon Ultra 100K for a buffer exchange. The sample was resuspended in 7% (w/w) sucrose buffer [5 mM Hepes/KOH (pH 7.6) containing 0.8 mM CaCl_2_, 96 mM NaCl, 2 mM KCl and 5 mM MgCl_2_]. The sample was then layered onto a 15% sucrose buffer and was ultracentrifuged at 163,000*g* for 2 hours at 10°C. The band containing the membrane fractions located at the top of the tube was collected and passed through a Superdex 200 column using an ÄKTA purifier system. The total amount of lipids in the fraction containing proteoliposomes was estimated using a LabAssay Phospholipid kit that quantified the amount of DOPC. For ion channel electrochemical recording, the lipid concentration was diluted to 3 μg/ml. Last, to confirm the OR-Orco in the liposomes, the sample was separated by SDS-PAGE using 12.5% SDS-PAGE gel and stained by oriole fluorescent gel stain. The protein concentrations after purification were estimated using a BSA standard curve (Image Lab, Bio-Rad).

### Current recording for octenol detection

The device was wired to a mounting system connected to a patch clamp amplifier (*31*). To form a lipid bilayer, 4 μl of *n*-decane containing DOPC:DOPE (20 mg/ml) at a 3:1 (w/w) ratio was loaded into the wells with no microslits. Then, 28 μl of buffer solution [5 mM Hepes/KOH (pH 7.6) containing 0.8 mM CaCl_2_, 96 mM NaCl, 2 mM KCl, and 5 mM MgCl_2_] was added to the wells with microslits. Subsequently, 24 μl of buffer containing proteoliposomes (lipid concentration: 3 μg/ml) was added to the wells with no microslits. The current was monitored at a sampling frequency of 5 kHz with a 1-kHz Bessel low-pass filter at a holding voltage of +60 mV. Octenol gas was generated by a standard gas generator (PD-1B-2, Gastec, Japan). Octenol gas was introduced to the device through a tube with the N_2_ carrier gas. The flow rate was monitored by a flow meter. To detect octenol in human breath, 1 liter of human breath was collected in Tedlar bags (AS ONE, Japan); then, octenol gas was injected into the bags to set the octenol concentration at 0.5 and 5 ppb. The gas samples were introduced to the device through a tube using a pump. A regulator maintained a constant flow rate of 0.2 liters/min.

## Supplementary Material

http://advances.sciencemag.org/cgi/content/full/7/3/eabd2013/DC1

Movie S1

Movie S2

Movie S3

Adobe PDF - abd2013_SM.pdf

Highly sensitive VOC detectors using insect olfactory receptors reconstituted into lipid bilayers

## SUPPLEMENTARY MATERIALS

Supplementary material for this article is available at http://advances.sciencemag.org/cgi/content/full/7/3/eabd2013/DC1

## Appendix

View/request a protocol for this paper from *Bio-protocol*.
