# Supplementary material for: Highly sensitive VOC detectors using insect olfactory receptors reconstituted into lipid bilayers
Source: Sci Adv. 2021 Jan 13;7(3):eabd2013. doi: 10.1126/sciadv.abd2013 (PMC7806217; doi:10.1126/sciadv.abd2013)
Supplement: http://advances.sciencemag.org/cgi/content/full/7/3/eabd2013/DC1 [file supp_7_3_eabd2013__index.html]

Science Advances | Science AdvancesAAASSearchScience AdvancesMenu

## Supplementary Materials

# Highly sensitive VOC detectors using insect olfactory receptors reconstituted into lipid bilayers

Tetsuya Yamada, Hirotaka Sugiura, Hisatoshi Mimura, Koki Kamiya, Toshihisa Osaki, Shoji Takeuchi

Download Supplement

**The PDF file includes:**

- Figs. S1 to S8
- Legends for movies S1 to S3

**Other Supplementary Material for this manuscript includes the following:**

- Movie S1
- Movie S2
- Movie S3

**Files in this Data Supplement:**

- Adobe PDF - abd2013\_SM.pdf
- abd2013\_Movie\_S1.mp4
- abd2013\_Movie\_S2.mp4
- abd2013\_Movie\_S3.mp4
