# Supplementary material for: Highly sensitive VOC detectors using insect olfactory receptors reconstituted into lipid bilayers
Source: Sci Adv. 2021 Jan 13;7(3):eabd2013. doi: 10.1126/sciadv.abd2013 (PMC7806217; doi:10.1126/sciadv.abd2013)
Supplement: http://advances.sciencemag.org/cgi/content/full/7/3/eabd2013/DC1 [file supp_7_3_eabd2013__1.pdf]

Science Advances
AAAS

advances.sciencemag.org/cgi/content/full/7/3/eabd2013/DC1

Supplementary Materials for

Highly sensitive VOC detectors using insect olfactory receptors reconstituted into lipid bilayers

Tetsuya Yamada, Hirotaka Sugiura, Hisatoshi Mimura, Koki Kamiya, Toshihisa Osaki, Shoji Takeuchi*

*Corresponding author. Email: takeuchi@hybrid.t.u-tokyo.ac.jp

Published 13 January 2021, *Sci. Adv.* **7**, eabd2013 (2021)
DOI: 10.1126/sciadv.abd2013

The PDF file includes:

Figs. S1 to S8
Legends for movies S1 to S3

Other Supplementary Material for this manuscript includes the following:

(available at advances.sciencemag.org/cgi/content/full/7/3/eabd2013/DC1)

Movies S1 to S3

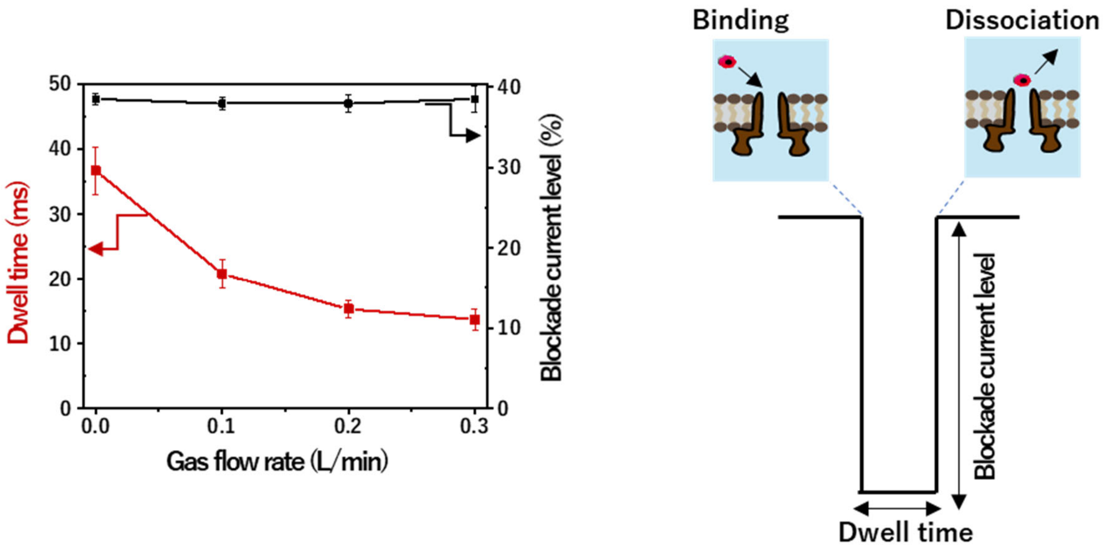

Fig. S1. Dwell time and blockade current levels against N_2 gas flowrate. The schematic on the right shows the current profiles of binding and dissociation between a nanopore and a blocker. Red: dwell time (ms). Black: blockade current level (%).

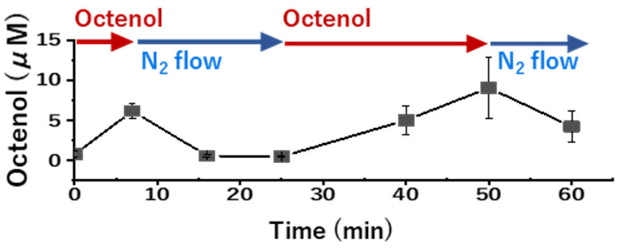

Fig. S2. Octenol concentration change in droplets estimated by gas chromatography. Red and blue arrows represent the introduction of octenol and N_2 gas flow to the microslits, respectively.

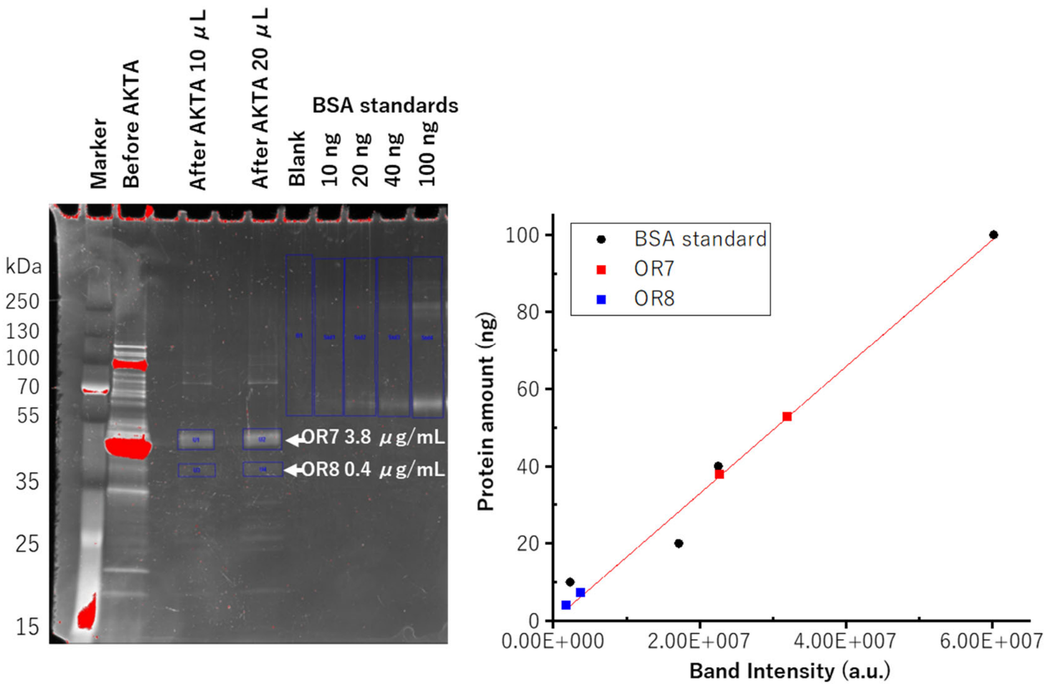

Fig. S3. SDS-PAGE analysis. This analysis was performed to confirm the incorporation of olfactory receptors (OR8) and their co-receptor proteins (OR7). The SDS-PAGE results for bovine serum albumin (BSA) at different concentrations were used as the reference standard. The concentrations of OR8 and OR7 were estimated based on the BSA standard curve.

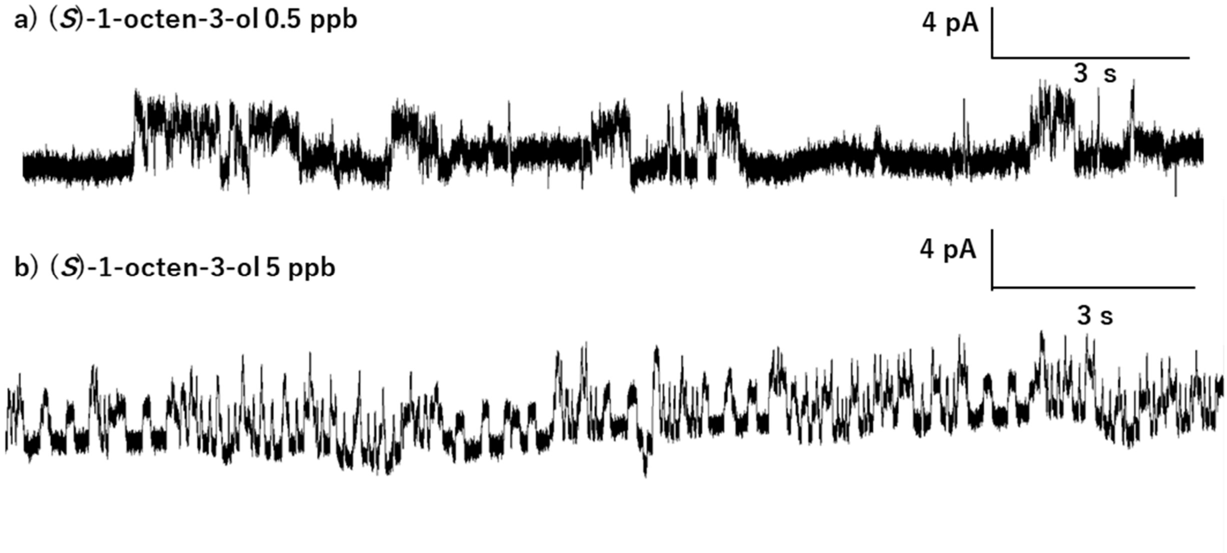

Fig. S4. Detection of octenol gas. Representative current traces of the OR-Orco in response to (a) 0.5 ppb (*S*)-1-octen-3-ol gas, open probability: 24%, and (b) 5 ppb (*S*)-1-octen-3-ol gas, open probability: 51%.

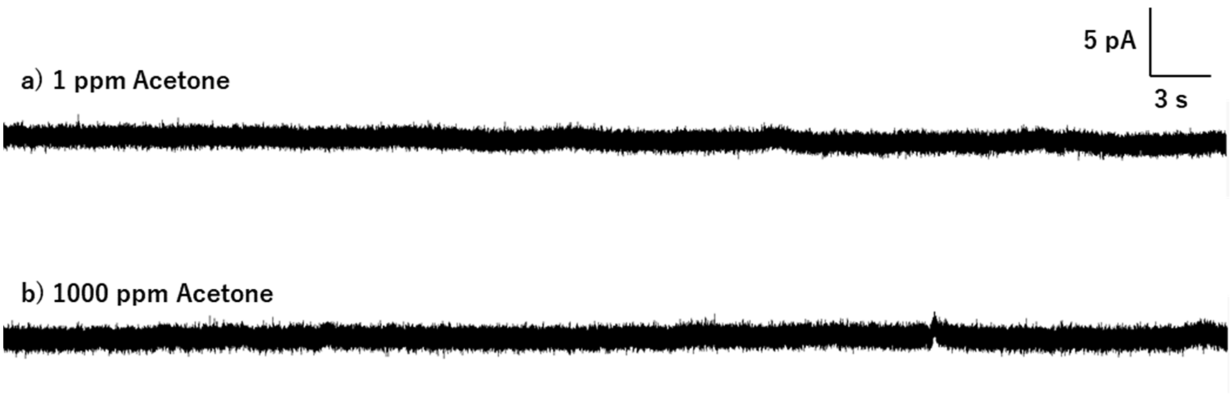

Fig. S5. Current trace for the olfactory receptor response to acetone. Representative current traces of the olfactory receptor in response to (a) 1 ppm acetone gas and (b) 1000 ppm acetone gas with N_2 carrier gas.

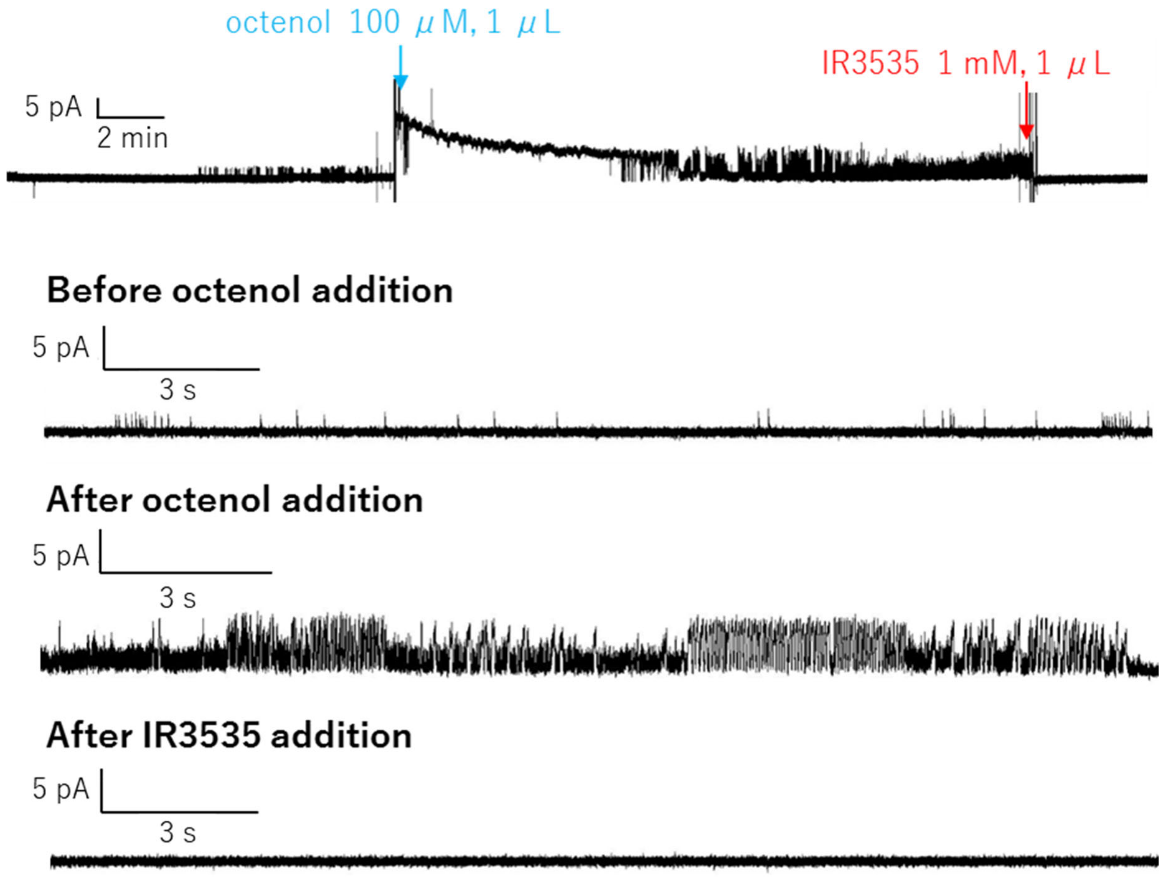

Fig. S6. Inhibitory experiment. IR3535 was added to inhibit olfactory receptor activity. Blue and red arrows indicate the addition of octenol and IR3535, respectively. The top-most figure shows a representative time course recording, and the lower three figures are enlarged diagrams of the three parts of the top-most figure separated by the blue and red arrows.

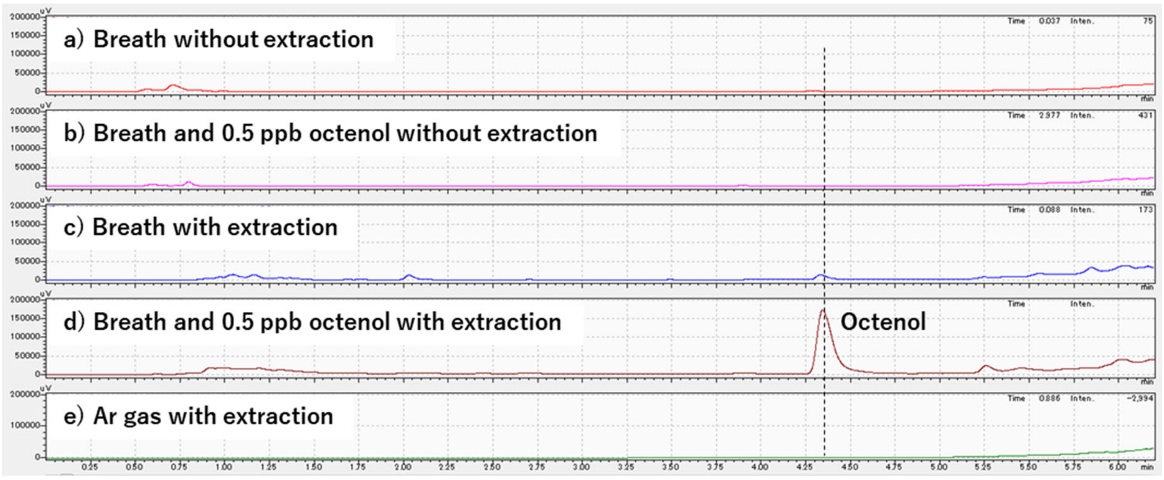

Fig. S7. GC analysis of breath. (a) Breath analysis without extraction; (b) breath and 0.5 ppb octenol; (c) breath subjected to extraction treatment, indicating that fresh human

breath contains 0.02 ppb octenol; (d) breath containing 0.5 ppb octenol subjected to extraction treatment; (e) Ar gas subjected to extraction treatment.

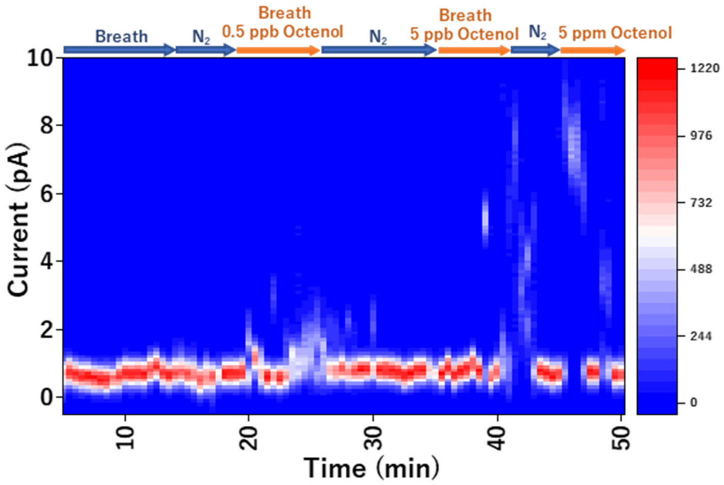

Fig. S8. Heat map of the current histogram over time. Blue and orange arrows indicate the introduction of N_2 gas (or breath) and breath mixed with octenol, respectively.

Movie S1: Introduction of ammonia gas into the 16 double-wells through the microchannel.

Phenolphthalein was added to the droplets as a pH indicator for the introduction of ammonia gas. The color change occurred because of the ammonia gas achieved the droplet through the microchannel.

Movie S2: Droplet mixing caused by the gas flow.

Microbeads (diameter: 45 µm) were added to the droplet. and their movement were observed under a microscope. When N_2 gas flow was initiated, the microbeads began to circulate.

Movie S3: Detection of octenol in human breath.

Human breath with octenol gas was introduced to the developed device, and the 16 current signals were monitored.
